# Topical Application of Frankincense Oil Extract Potently Ameliorates Psoriasis-like Dermatitis in Mice via Anti-Inflammatory and Skin Barrier-Protective Effects

**DOI:** 10.3390/ijms27062629

**Published:** 2026-03-13

**Authors:** Wen-Jing Li, Li-Ying Wen, Yu-Sang Li, He-Bin Tang

**Affiliations:** Lab of Hepatopharmacology and Ethnopharmacology, School of Pharmaceutical Sciences, South-Central Minzu University, No. 182, Minyuan Road, Wuhan 430074, China; 2024110499@mail.scuec.edu.cn (W.-J.L.); 2022110508@mail.scuec.edu.cn (L.-Y.W.)

**Keywords:** psoriasis, frankincense and its active ingredients, TRPV3, skin barrier, hyperproliferation

## Abstract

Frankincense, a traditional Chinese medicinal resin with well-documented skin barrier-protective and anti-inflammatory properties, has elusive underlying mechanisms in psoriasis-like dermatitis. This study aimed to elucidate its therapeutic potential and molecular targets by investigating frankincense oil extract (FOE) and three key constituents (linalool, α-pinene and 1-octanol) in a classic imiquimod-induced murine psoriasis model, with clinical first-line topical drugs (calcipotriol, tapinarof and dithranol) used as positive controls. Phenotypically, FOE and its constituents significantly ameliorated core psoriasis symptoms (desquamation, erythema, epidermal thickening and splenomegaly) at an efficacy comparable to that of positive controls. FOE suppressed epidermal hyperproliferation and dermal inflammatory infiltration, attenuated the abnormally elevated epidermal expression of TRPV3, β-catenin and COX-2, and increased the expression of the barrier protein K10. Taken together, these findings suggest that FOE restores impaired epidermal barrier function by regulating TRPV3, β-catenin, COX-2 and K10 expression, providing a novel mechanistic basis for the clinical application of traditional frankincense in psoriasis and identifying promising targets for antipsoriatic-drug development.

## 1. Introduction

Psoriasis is an immune-mediated chronic proliferative skin disorder characterized by epidermal keratinocyte hyperproliferation and dermal inflammatory cell infiltration [1]. Psoriasis has a global lifetime prevalence of 1.12% in adults and 0.14% in East Asian adults, and the scales and erythema associated with psoriatic lesions severely impair patients’ quality of life [2]. Currently, clinical treatments for psoriasis mainly include vitamin D3 analogs, glucocorticoids, and antikeratinizing agents, which act primarily by inhibiting epidermal cell hyperproliferation and inducing differentiation [3,4]. However, these agents are associated with limited efficacy, frequent recurrence, drug resistance, and adverse effects following long-term use, highlighting the urgent need for safe, effective, and easily applicable topical therapies.

Traditional Chinese medicine has a long history of using emollient agents for psoriasis management, with research attention increasingly focused on natural products that enhance skin barrier function [5]. Frankincense, a medicinal resin documented in the Chinese Pharmacopoeia, promotes blood circulation, reduces edema, and facilitates tissue regeneration [6]. This medicinal resin has been widely applied topically in dermatological practice for treating various skin disorders [7]. Previous studies have demonstrated that *Boswellia carteri* Birdw. resin extract and its major bioactive constituents (linalool, α-pinene, and 1-octanol) accelerate cutaneous wound healing by inhibiting the β-catenin/cyclooxygenase-2 (COX-2) pathway to downregulate inflammatory mediators and by promoting normal keratinocyte differentiation to restore epidermal barrier structure [7,8,9].

Notably, transient receptor potential vanilloid 3 (TRPV3), a temperature-sensitive calcium channel, plays a crucial role in regulating epidermal homeostasis and skin barrier function [10,11]. Aberrant TRPV3 activation contributes to skin hypersensitivity, which in turn induces abnormal keratinocyte hyperproliferation and the subsequent disruption of the skin barrier, key pathological features of psoriasis [12,13]. In the present study, the therapeutic efficacy of frankincense in imiquimod (IMQ)-induced psoriasis-like dermatitis, its distinct advantages over clinically approved positive control agents in psoriasis prevention and treatment, and its active constituent-mediated effects were systematically investigated.

## 2. Results

### 2.1. Clinical Drugs and Inhibitors Attenuate IMQ-Induced Psoriasis-like Inflammation

**Figure 1:**
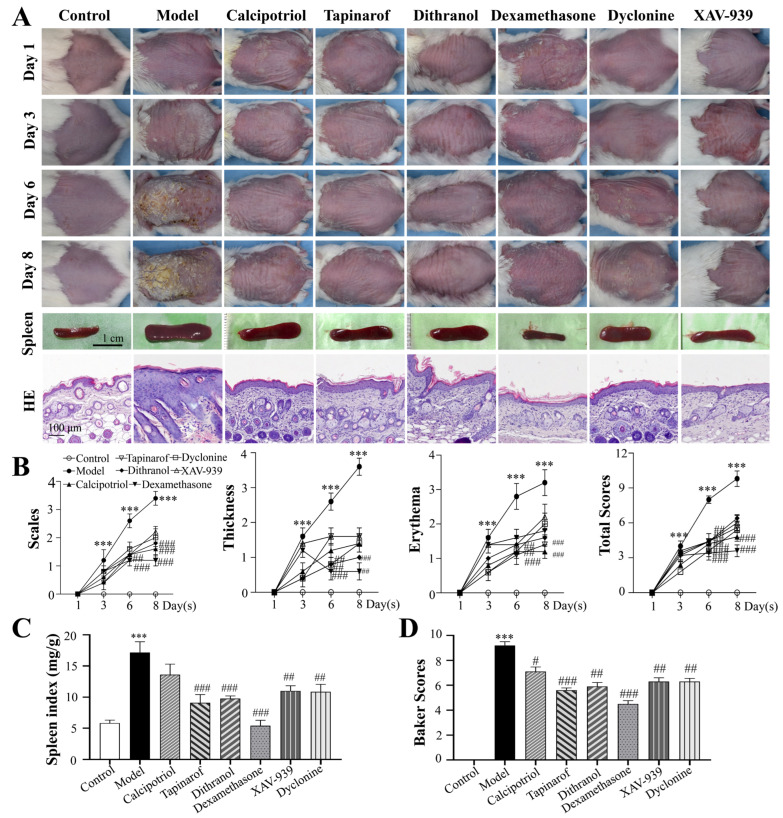
Effects of clinically used drugs and related inhibitor treatments on IMQ-induced psoriasis in mice. (**A**) Mice were left untreated or subjected to 5% IMQ, and the appearance of the dorsal skin was examined via H&E staining for pathological changes. Scale bars: 100 μm. (**B**) PASI scores were recorded on days 0, 3, 6, and 8 and the cumulative scores of scaling, erythema, and thickness were measured. (**C**) Spleen index. (**D**) Baker scores. The experimental data are presented as the mean ± SEM. Compared with the control, *** *p* < 0.001. Compared with the model group, # *p* < 0.05; ## *p* < 0.01; ### *p* < 0.001.

Three clinically widely used antipsoriatic agents (calcipotriol, tapinarof, and dithranol) and three specific inhibitors (XAV-939, a β-catenin inhibitor; dexamethasone, a COX-2 inhibitor; and dyclonine, a TRPV3 inhibitor) were selected for this study. As shown in Figure 1A, compared with the control group, the topical application of IMQ significantly induced desquamation, erythema, and dermal thickening from day 6 onward, confirming the successful establishment of the psoriasis-like murine model. In contrast, substantial attenuation of these phenotypic abnormalities and a reduction in disease severity scores were achieved following intervention with the selected pharmacological agents or specific inhibitors.

Notably, IMQ treatment triggered a systemic inflammatory response characterized by splenomegaly, a typical pathological feature of this murine model. IMQ-induced splenomegaly was significantly alleviated by all clinical drugs and specific inhibitors. Interestingly, compared with the control group, long-term dexamethasone treatment resulted in splenic atrophy, with significantly lower weight and size.

Histopathologically, compared with those in the IMQ-induced model group, the epidermal thickness and dermal inflammatory cell infiltration were significantly reduced in all treatment groups. Additionally, the dermal fiber content was further decreased by dexamethasone treatment, indicating a more comprehensive regulatory effect on skin pathology.

As shown in Figure 1B, significantly lower inhibitory rates against IMQ-induced epidermal hyperproliferation were observed in the calcipotriol, tapinarof, and dithranol ointment-treated groups (36.75 ± 1.83%, 27.14 ± 3.76%, and 35.26 ± 3.74%, respectively). These findings are consistent with the established role of IMQ in inducing epidermal hyperproliferation in psoriasis-like models. The effects of related inhibitors on IMQ-induced psoriatic lesions were further evaluated. Compared with that in the model group, lesion severity was significantly reduced by treatment with dexamethasone, XAV-939, and dyclonine, whose inhibitory effects on IMQ-induced pathological changes were 42.87 ± 4.18%, 36.15 ± 5.27%, and 28.90 ± 1.82% respectively. Among these agents, dexamethasone had the most prominent therapeutic effect.

### 2.2. Therapeutic Efficacy of FOE and Its Active Ingredients in IMQ-Induced Psoriasis-like Inflammation

**Figure 2:**
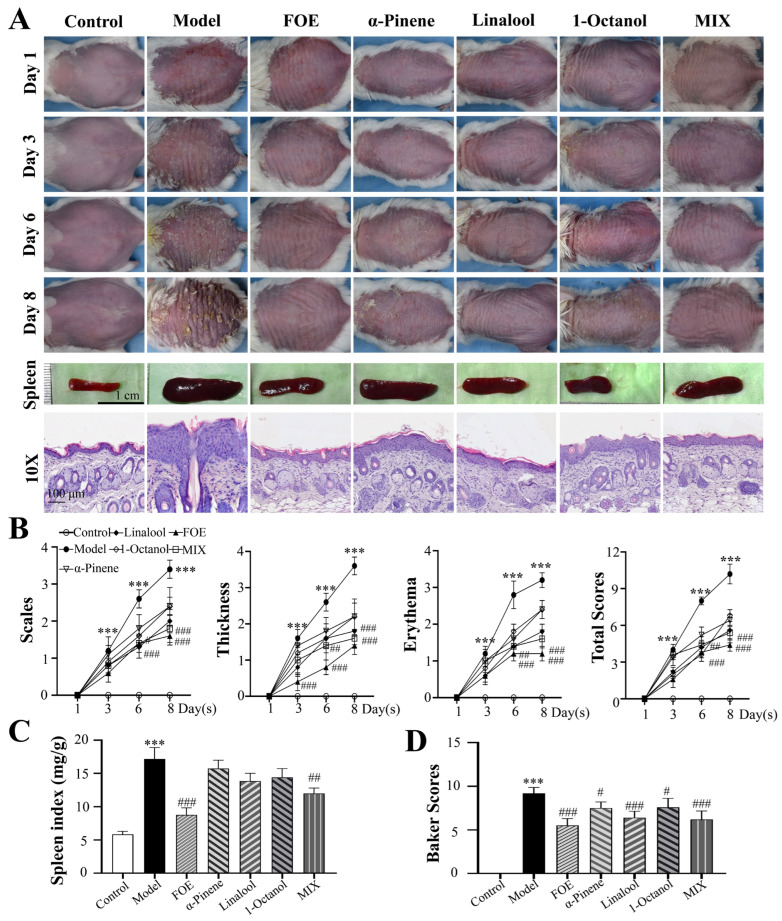
Effects of treatment with FOE and its active ingredients on IMQ-induced psoriasis in mice. (**A**) Mice were left untreated or subjected to 5% IMQ, and the appearance of the dorsal skin was examined via H&E staining for pathological changes. Scale bars: 100 μm. (**B**) PASI scores were recorded on days 0, 3, 6, and 8 and the cumulative scores of scaling, erythema, and thickness were measured. (**C**) Spleen index. (**D**) Baker scores. The experimental data are presented as the mean ± SEM. Compared with the control, *** *p* < 0.001. Compared with the model group, # *p* < 0.05; ## *p* < 0.01; ### *p* < 0.001.

The present study aimed to evaluate the therapeutic potential of FOE in an IMQ-induced murine model of psoriasis-like dermatitis (Figure 2). Compared with the IMQ-induced model group, the topical application of FOE significantly ameliorated disease symptoms, characterized by reduced symptom severity, improved skin texture, and decreased erythema intensity. In contrast, although individual bioactive constituents of FOE (linalool, α-pinene, and 1-octanol) effectively alleviated dermal thickening, their effects on erythema were less pronounced than those of FOE. Quantitative analysis using the Psoriasis Area and Severity Index (PASI) confirmed that a significant reduction in erythema severity was achieved with FOE treatment, whereas no statistically significant improvement in this parameter was observed with individual bioactive constituents.

On day 8 of treatment, histopathological analysis revealed significant improvements in epidermal thickness and reduced inflammatory cell infiltration in mice treated with FOE, linalool, α-pinene, 1-octanol, or the three active ingredients in combination compared with those in the IMQ-induced model group. Specifically, the inhibitory rates against IMQ-induced epidermal hyperplasia were 48.94 ± 3.61%, 57.39 ± 1.84%, 66.61 ± 2.59%, 66.96 ± 4.28% and 65.39 ± 4.01% for the aforementioned treatments, respectively. The corresponding inhibitory rates against inflammatory cell infiltration were 23.20 ± 1.12%, 33.26 ± 3.27%, 32.91 ± 2.34%, 43.71 ± 4.97% and 30.76 ± 2.15%, respectively.

These findings offer compelling evidence that topical FOE intervention effectively attenuates both the clinical manifestations and histopathological abnormalities associated with IMQ-induced psoriasis-like dermatitis. Notably, IMQ-induced splenomegaly was significantly reduced by topical FOE application, and a similar splenomegaly-alleviating effect was observed with the active constituents of FOE. However, the therapeutic efficacy of individual active constituents was less pronounced than that of FOE.

### 2.3. Effects of Clinical Drugs and Inhibitors on Epidermal Keratinocytes in IMQ-Induced Psoriasis Model Mice

In this study, H&E staining was performed to obtain clear epidermal structural images. This allowed analysis of differences in the type and number of epidermal keratinocytes among groups, as well as investigation of the effects of clinically used drugs and specific inhibitors on keratinocyte proliferation, differentiation, and migration in the epidermis of IMQ-induced psoriatic mice.

**Figure 3:**
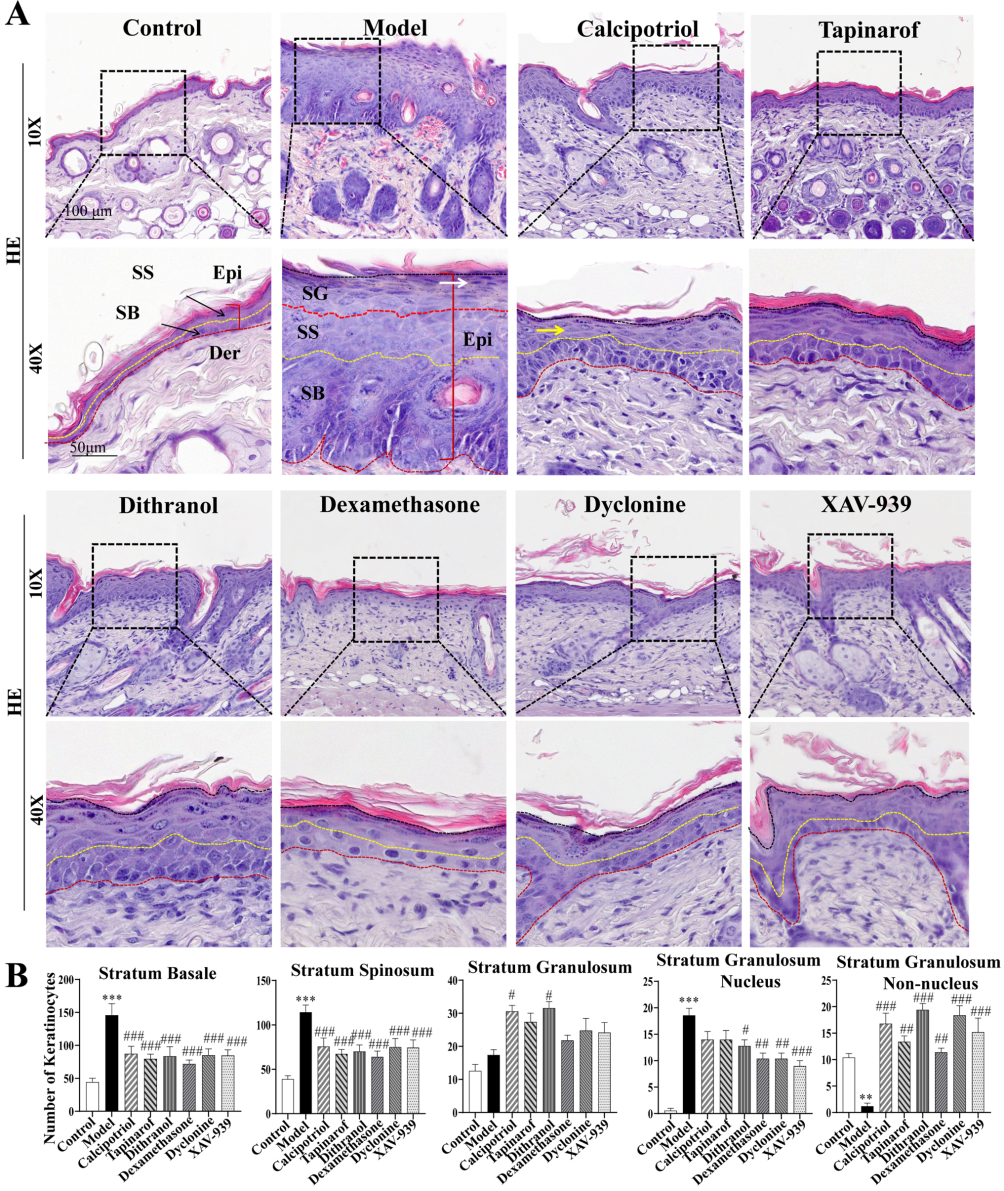
Effects of drug/inhibitor treatments on histopathological changes in the skin of IMQ-induced psoriasic mice. (**A**) Histopathological changes in the backs of the skin at 8 days after IMQ treatment. Epi, Der, SB, SS, and SG represent Epidermis, Dermis, Stratum Basale, Stratum Spinosum, and Stratum Granulosum, respectively. Black arrows point to Stratum Basale and Stratum Spinosum. White arrows indicate parakeratotic keratinocytes, and yellow arrows indicate differentiated granular layer cells. Scale bars: 100 μm, 50 μm. (**B**) Number of keratinocytes in the epidermis. The experimental data are presented as the mean ± SEM. Compared with the control, ** *p* < 0.01; *** *p* < 0.001. Compared with the model group, # *p* < 0.05; ## *p* < 0.01; ### *p* < 0.001.

As shown in Figure 3, compared with the control group, the IMQ-induced model group exhibited a significant increase in the number of proliferative stratum basale (SB) cells (*p* < 0.001) and stratum spinosum (SS) cells (*p* < 0.001), whereas the number of differentiated stratum granulosum (SG) cells did not significantly differ (*p* > 0.05). These findings confirm that IMQ-induced pathology accelerates epidermal keratinocyte proliferation without affecting differentiation, contributing to psoriatic epidermal hyperplasia.

Treatment with clinically available drugs (calcipotriol, tapinarof, and dithranol) reduced the number of stratum spinosum cells and increased the number of stratum granulosum cells. Additionally, these drugs decreased the proportion of nucleated cells in the stratum granulosum and significantly increased the number of anucleated cells in this layer. These results indicate that the three drugs inhibit abnormal keratinocyte proliferation, induce keratinocyte differentiation, accelerate cell migration, and restore the normal proportional distribution of epidermal layers. Furthermore, the proportion of differentiated cells in the drug-treated groups tended to be greater than that in the control group, suggesting that drug intervention alleviates epidermal hyperkeratosis by increasing the differentiation rate of keratinocytes.

In addition, TRPV3 inhibitors and β-catenin inhibitors also increased the number of stratum granulosum cells to a certain extent. These observations support the notion that inhibition of TRPV3 or β-catenin signaling may contribute to the suppression of excessive keratinocyte proliferation in psoriasis-like pathology.

### 2.4. Effects of FOE and Its Active Ingredients on Epidermal Keratinocytes in IMQ-Induced Psoriasis Model Mice

**Figure 4:**
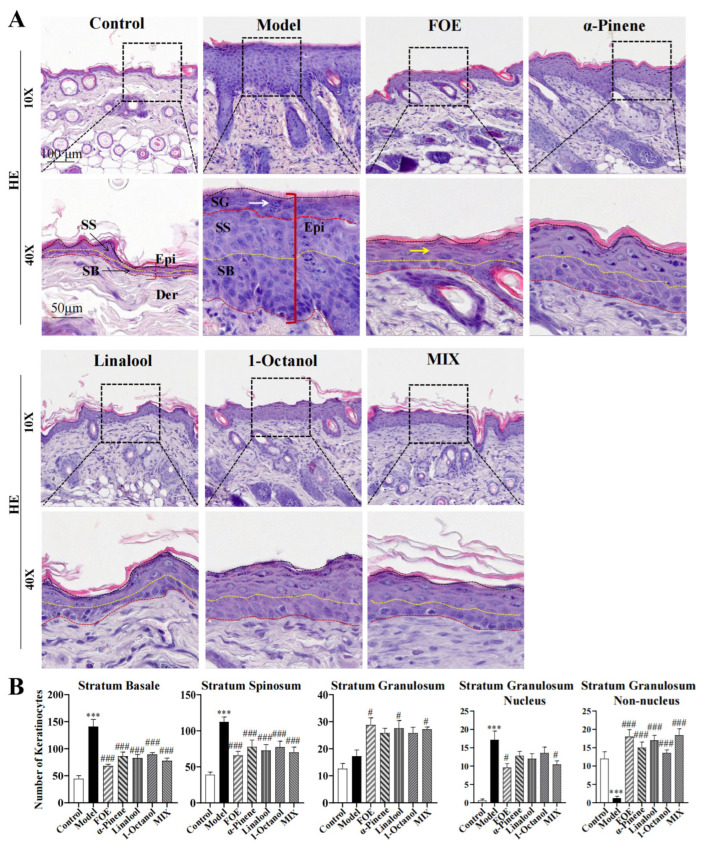
Effects of FOE treatment on the histopathological changes in the skin of IMQ-induced psoriasic mice. (**A**) Histopathological changes in the backs of the skin at 8 days after IMQ treatment. Epi, Der, SB, SS, and SG stands for Epidermis, Dermis, Stratum Basale, Stratum Spinosum, and Stratum Granulosum, respectively. Black arrows indicate Stratum Basale and Stratum Spinosum. White arrows indicate parakeratotic keratinocytes, and yellow arrows indicate differentiated granular layer cells. Scale bars: 100 μm, 50 μm. (**B**) Number of keratinocytes in the epidermis. The experimental data are presented as the mean ± SEM. Compared with the control, *** *p* < 0.001. Compared with the model group, # *p* < 0.05; ### *p* < 0.001.

By comparing the numbers of different epidermal cell subsets between the IMQ-induced model group and the FOE-treated group (Figure 4), we found that the numbers of SB cells and SS cells in the dorsal epidermis significantly decreased in the FOE group, whereas the numbers of SG cells markedly increased. These observations indicate that FOE enhances the differentiation capacity of epidermal keratinocytes.

Moreover, the proportion of differentiated cells in the FOE group tended to be greater than that in the control group, with fewer nucleated cells in the stratum granulosum. Additionally, the number of anucleated cells in the stratum granulosum significantly increased in the FOE group. These findings suggest that FOE alleviates epidermal hyperkeratosis by promoting keratinocyte differentiation, thereby facilitating the normalization of epidermal structure.

Additionally, individual active constituents of FOE moderately increased the number of granular layer keratinocytes in the dorsal skin. Notably, the combined formulation (MIX) of these active constituents substantially reduced the total epidermal cell number, with a specific decrease in stratum spinosum cells. These results support the notion that the active constituent mixture of FOE exerts a potent effect on normalizing the epidermal hyperplasia characteristic of psoriatic lesions.

### 2.5. Clinical Drugs and Inhibitors Repair Skin Barrier by Inhibiting TRPV3 Overactivation and Enhancing K10 Expression

**Figure 5:**
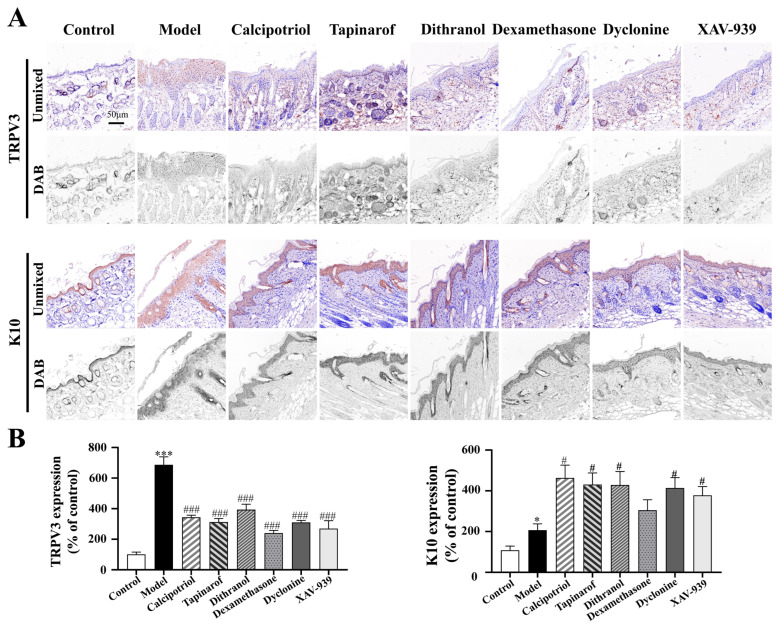
Immunohistochemical staining of TRPV3 and K10 in murine skin tissues and quantitative representative images. (**A**) Representative images of immunohistochemical staining for TRPV3 and K10 in the skin of psoriasis mice in the groups treated clinically with drug groups and related inhibitor groups. (**B**) Quantitative multispectral image of TRPV3 and K10 expression in the groups treated clinically with drugs and related inhibitor groups. Two-way ANOVA was used to express the experimental data as the mean ± SEM. Compared with the control, * *p* < 0.05; *** *p* < 0.001. Compared with the model group, # *p* < 0.05; ### *p* < 0.001. Scale bar, 50 µm.

As illustrated in Figure 5, quantitative analysis was performed with reference to the control group. Compared with the control group, the IMQ-induced model group exhibited significantly elevated TRPV3 protein expression (686 ± 53%, *p* < 0.001) and markedly reduced keratin 10 (K10) expression (206 ± 32%, *p* < 0.05), confirming the successful establishment of the psoriasis-like model.

Notably, compared with the model group, all therapeutic intervention groups showed significant reduction in TRPV3 expression: calcipotriol (343 ± 14% of the control, *p* < 0.001), tapinarof (311 ± 24% of the control, *p* < 0.001), and dithranol (393 ± 36% of the control, *p* < 0.001). Conversely, K10 expression was significantly enhanced in these groups compared with that in the model group: calcipotriol (463 ± 64% of the control, *p* < 0.05), tapinarof (430 ± 57% of the control, *p* < 0.05), and dithranol (428 ± 66% of the control, *p* < 0.05).

In support of these findings, treatment with specific inhibitors, such as dyclonine, XAV-939, and dexamethasone, also resulted in similar regulatory effects: significantly suppressed TRPV3 expression and increased K10 expression compared with those in the model group.

Collectively, these results suggest that clinically used antipsoriatic drugs, including calcipotriol, tapinarof and dithranol, may ameliorate psoriasis by inhibiting the pathological overexpression of TRPV3 and enhancing K10 expression, a mechanism that contributes to skin barrier repair.

### 2.6. FOE and Its Active Components Repair the Skin Barrier by Regulating TRPV3 and K10 Expression

**Figure 6:**
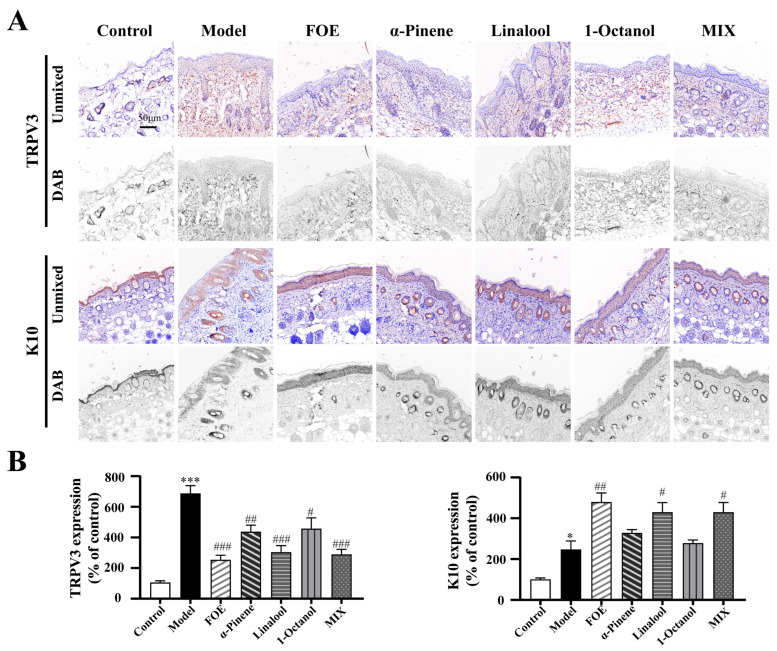
Immunohistochemical staining of TRPV3 and K10 in murine skin tissues and quantitative representative images. (**A**) Representative images of immunohistochemical staining for TRPV3 and K10 in the skin of psoriasis mice in the FOE and active ingredient groups. (**B**) Quantitative multispectral image of TRPV3 and K10 expression in the FOE and active ingredient groups. Two-way ANOVA was used to express the experimental data as the mean ± SEM. Compared with the control, * *p* < 0.05; *** *p* < 0.001. Compared with the model group, # *p* < 0.05; ## *p* < 0.01; ### *p* < 0.001. Scale bar, 50 µm.

As illustrated in Figure 6, the protein expression levels were normalized to those in the control group. Compared with the control group, the IMQ-induced model group exhibited significant elevation in TRPV3 (686 ± 53% of the control, *p* < 0.001) and marked reduction in K10 (246 ± 42% of the control, *p* < 0.05), confirming successful establishment of the psoriasis-like model.

With respect to TRPV3 regulation, all treatment groups showed significant downregulation relative to the model group: FOE (251 ± 32%, *p* < 0.001), the combined formulation (MIX; 289 ± 34%, *p* < 0.001), linalool (303 ± 44%, *p* < 0.001), α-pinene (435 ± 46%, *p* < 0.01), and 1-octanol (456 ± 72%, *p* < 0.05). Among these, FOE exerted the most potent inhibitory effect on TRPV3 overexpression.

In contrast, K10 expression was significantly elevated in the FOE-treated group compared with that in the model group (478 ± 45%, *p* < 0.01), which was greater than that in the MIX (428 ± 49%, *p* < 0.05) and linalool (448 ± 35% of the control, *p* < 0.05) groups. However, compared with the model group, α-pinene (327 ± 17%, *p* > 0.05) and 1-octanol (277 ± 16%, *p* > 0.05) failed to significantly increase K10 expression.

Collectively, these results demonstrate that FOE and its active constituents regulate the expression of TRPV3 and K10 in psoriatic lesions. Specifically, they inhibit the pathological overexpression of TRPV3 and enhance K10 expression, a mechanism that may underpin their therapeutic efficacy in ameliorating psoriasis-like pathology.

### 2.7. Clinically Used Drugs and Inhibitors Suppress Psoriatic Phenotypes by Inhibiting β-Catenin and COX-2 Overactivation

**Figure 7:**
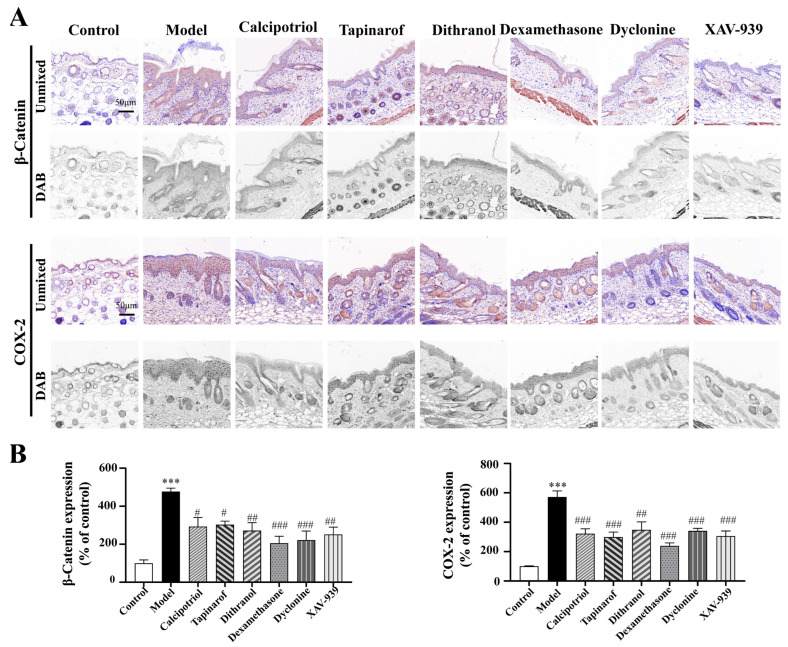
Immunohistochemical staining of β-catenin and COX-2 in murine skin tissues and quantitative representative images. (**A**) Representative images of immunohistochemical staining for β-catenin and COX-2 in the skin of psoriasis mice in the groups treated clinically with drugs and related inhibitors. (**B**) Quantitative multispectral images of β-catenin and COX-2 expression in the groups treated with clinical drugs and related inhibitors. Two-way ANOVA was used to express the experimental data as the mean ± SEM. Compared with the control, *** *p* < 0.001. Compared with the model group, # *p* < 0.05; ## *p* < 0.01; ### *p* < 0.001. Scale bar, 50 µm.

As illustrated in Figure 7, the protein expression levels were normalized to those in the control group. Compared with the control, the IMQ-induced untreated model group exhibited significant overexpression of β-catenin (456 ± 5% of the control, *p* < 0.001) and COX-2 (572 ± 41% of the control, *p* < 0.001), confirming the successful establishment of the psoriasis-like model.

Compared with the model group, all the therapeutic intervention groups showed significant reduction in β-catenin and COX-2 expression. Calcipotriol treatment markedly reduced the levels of target proteins, resulting in values of 292 ± 67% (*p* < 0.05) and 322 ± 33% (*p* < 0.001) for β-catenin and COX-2, respectively; tapinarof administration resulted in values of 303 ± 19% (*p* < 0.05) and 299 ± 35% (*p* < 0.001) for β-catenin and COX-2, respectively. Dithranol-treated samples presented intermediate expression levels (β-catenin: 272 ± 42%, *p* < 0.01; COX-2: 348 ± 55%, *p* < 0.001). Notably, dexamethasone intervention resulted in the most pronounced attenuation of both molecular markers within epidermal compartments (β-catenin: 205 ± 37%, *p* < 0.001; COX-2: 239 ± 20%, *p* < 0.001).

Collectively, these findings demonstrate that established antipsoriatic agents and specific inhibitors mediate their anti-inflammatory effects on psoriatic pathogenesis by inhibiting the pathological overexpression of β-catenin and COX-2.

### 2.8. FOE and Its Active Components Alleviate Psoriatic Phenotypes by Inhibiting β-Catenin and COX-2 Overactivation

**Figure 8:**
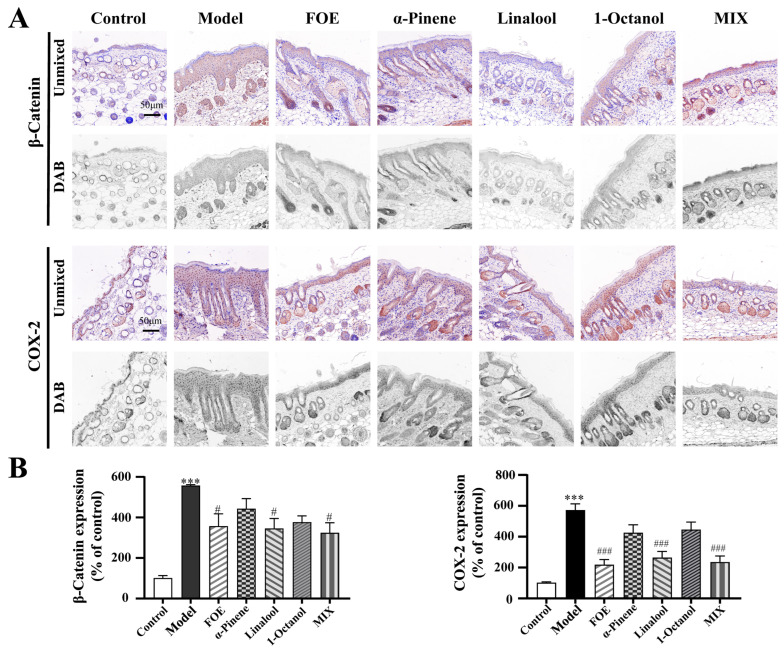
Immunohistochemical staining of β-catenin and COX-2 in murine skin tissues and quantitative representative images. (**A**) Representative images of immunohistochemical staining for β-catenin and COX-2 in the skin of psoriasis mice in the FOE and active ingredient groups. (**B**) Quantitative multispectral image of β-catenin and COX-2 expression in the FOE and active ingredient groups. Two-way ANOVA was used to express the experimental data as mean ± SEM. Compared with the control, *** *p* < 0.001. Compared with the model group, # *p* < 0.05; ### *p* < 0.001. Scale bar, 50 µm.

As illustrated in Figure 8, immunohistochemical analysis was performed, and the protein expression levels were normalized to those in the control group. Compared with the control group, the IMQ-induced model group exhibited significant overexpression of β-catenin (556 ± 6% of the control, *p* < 0.001) and COX-2 (572 ± 41% of the control, *p* < 0.001), confirming successful establishment of the psoriasis-like model.

Compared with the model group, all the treatment groups showed significant attenuation of β-catenin and COX-2. Specifically, FOE administration resulted in β-catenin and COX-2 expression levels of 356 ± 62% (*p* < 0.05) and 218 ± 35%, respectively (*p* < 0.001), whereas the combined formulation (MIX) resulted in values of 323 ± 51% (*p* < 0.05) and 235 ± 41%, respectively (*p* < 0.001).

The individual active constituents exhibited varying degrees of inhibitory efficacy. Linalool had significant inhibitory effects, with β-catenin expression at 345 ± 50% (*p* < 0.05 versus the model group) and COX-2 expression at 264 ± 41% (*p* < 0.001 versus the model group). In contrast, α-pinene (β-catenin: 442 ± 51%, *p* > 0.05; COX-2: 425 ± 52%, *p* > 0.05) and 1-octanol (β-catenin: 416 ± 50%, *p* > 0.05; COX-2: 445 ± 50%, *p* > 0.05) failed to achieve statistical significance relative to the model group.

Collectively, these quantitative findings support the hypothesis that FOE and its active constituents mitigate inflammatory responses in psoriatic pathogenesis by inhibiting the pathological overexpression of β-catenin and COX-2.

## 3. Discussion

The effects and potential mechanisms of FOE and its active ingredients on IMQ-induced acute psoriasis-like dermatitis were explored in the present study. Experimental results demonstrated that FOE and its active ingredients significantly ameliorated psoriasis-like manifestations, including scaling, erythema, skin thickening, splenomegaly, epidermal hyperplasia, and dermal inflammatory cell infiltration. Furthermore, FOE effectively downregulated the overexpression of the TRPV3, β-catenin, and COX-2 proteins induced by IMQ stimulation, highlighting its potential as a promising therapeutic candidate for psoriasis management.

The therapeutic efficacy of the aforementioned inhibitors and FOE was evaluated across multiple indices (Supplementary Figure S1). Compared with the IMQ-induced model group, the FOE-treated group exhibited the highest recovery rate of skin lesions (45%), followed by the dexamethasone group (43%), while the other groups showed lower recovery rates. These results indicate that compared with the other agents, FOE has superior therapeutic effects on psoriasis-like dermatitis, with the additional advantage of avoiding splenic inhibitory side effects.

Epidermal hyperplasia and dermal inflammatory cell infiltration are well-recognized core pathological features of psoriasis [14]. In the present study, the therapeutic potential of several clinically approved antipsoriatic agents assessed, including the corticosteroid dexamethasone, the vitamin D analog calcipotriol, the aryl hydrocarbon receptor modulator tapinarof, and anthralin (dithranol), was assessed [15,16,17]. These agents are known to reduce abnormal skin thickening, desquamation, and dermal inflammatory cell accumulation. Notably, the weight and size of the spleen, a key immune organ involved in psoriasis progression, typically increase in response to disease severity. Compared with normal control mice, mice treated with calcipotriol, tapinarof, or dithranol still presented with splenomegaly, suggesting that these three agents lack significant immunomodulatory effects and thus may not achieve complete disease resolution.

In contrast, topical dexamethasone treatment led to skin and spleen atrophy in mice. As a potent immunosuppressant, dexamethasone alleviates inflammation by suppressing the immune system; however, long-term use can impair splenic function and inhibit cutaneous collagen synthesis, resulting in skin fragility. Although dexamethasone temporarily relieves excessive skin inflammation by suppressing spleen function, long-term dependence may impair the ability of the immune system to support skin repair, ultimately hindering psoriasis recovery. In sharp contrast, topical FOE administration markedly alleviated psoriasis-like symptoms in IMQ-induced mice without inducing dermal or splenic atrophy. Compared with the model group, FOE treatment significantly reduced epidermal thickness, inhibited dermal inflammatory cell infiltration, and alleviated splenomegaly, indicating that FOE possesses both anti-inflammatory and immunomodulatory properties in the context of psoriasis-like dermatitis. Overall, FOE avoids the major safety risks associated with long-term conventional psoriasis therapies, such as immune dysfunction, dermal atrophy, and local irritation. While long-term toxicity studies in larger animal models are still needed to fully confirm its safety, our current results support FOE’s potential as a safer option for long-term topical psoriasis treatment.

Accumulating evidence has demonstrated that TRP channels, which are localized in keratinocytes and neuronal terminals, mediate proliferative and immunomodulatory pathways associated with chronic cutaneous disorders such as psoriasis and atopic dermatitis vulgaris [18,19]. Among these channels, TRPV3, a vanilloid subtype of the TRP superfamily, is functionally expressed in epidermal keratinocytes [20]. Properly regulated TRPV3-mediated signaling is critical for maintaining epidermal barrier homeostasis [21], whereas aberrant TRPV3 activation contributes to skin barrier disruption, epidermal hyperplasia, inflammation, and pruritus (key pathological features of psoriasis) [22,23]. In the present study, TRPV3 was overexpressed in the skin lesions of IMQ-induced mice and was predominantly localized to epidermal keratinocytes, particularly proliferative keratinocytes. Compared with model group, mice treated with dyclonine (a TRPV3 inhibitor) presented reduced psoriasis-like inflammation severity and decreased keratinocyte TRPV3 expression, supporting the notion that pharmacological blockade of TRPV3 signaling represents a promising therapeutic strategy for psoriasis. Importantly, treatment with frankincense oil extract (FOE) yielded analogous effects, indicating that the therapeutic actions of FOE against psoriasis are likely mediated by suppressing TRPV3 expression, restoring skin barrier integrity, attenuating inflammatory responses, and alleviating pruritus.

The expression and function of keratins in skin keratinocytes have been extensively investigated [24]. K10, a well-established marker of keratinocyte differentiation, is essential for epidermal cell growth and differentiation [25]. In psoriasis pathogenesis, reduced K10 expression in the upper basal layer of epidermal cells contributes to abnormal epidermal proliferation and differentiation [26]. In the current study, clinical topical agents (calcipotriol, tapinarof, and dithranol) reduced epidermal hypertrophy and thickened the granular layer in murine lesions while increasing K10 expression in the spinous and granular layers, indicating enhanced keratinocyte differentiation. Similar results were observed following FOE intervention, suggesting that FOE attenuates psoriatic epidermal hyperplasia by enhancing K10 expression, thereby promoting epidermal structural normalization.

In addition to epidermal barrier restoration, epidermal proliferation and dermal inflammation were key focuses of this study. Epidermal hyperplasia in psoriasis is often accompanied by the accumulation of dermal inflammatory factors [27], with abnormally elevated β-catenin expression in proliferating epidermal cells [28]. As a critical structural protein involved in cutaneous morphogenesis and tissue repair, β-catenin exhibits distinct spatial expression patterns across epidermal and dermal compartments [29]. During cutaneous inflammatory responses, β-catenin is translocated from the cytoplasm to the membrane and nucleus, inducing COX-2 production and the formation of a positive feedback loop that amplifies β-catenin levels. Previous studies from our laboratory have demonstrated that FOE exerts anti-inflammatory effects on dermal tissues by suppressing β-catenin/COX-2 signaling. Consistent with these findings, IMQ-induced mice presented markedly elevated COX-2 and β-catenin levels in epidermal tissue. Intervention with XAV-939 (a β-catenin inhibitor) resulted in significant improvement in psoriasis-like skin lesions and the concurrent reduction in β-catenin expression, supporting the therapeutic hypothesis that modulation of β-catenin signaling mitigates pathological keratinocyte proliferation in psoriasis. Similarly, although psoriatic skin lesion repair was promoted and COX-2 expression was significantly diminished by dexamethasone treatment, skin and spleen atrophy was induced. These findings indicate that although inflammation inhibition alleviates psoriasis-like symptoms, nonspecific immunosuppression may impair immune function and compromise long-term healing. In contrast, topical FOE treatment achieved high therapeutic efficacy without inducing skin or spleen atrophy, accompanied by marked attenuation of both β-catenin and COX-2 expression. These findings suggest that the therapeutic effects of FOE and its bioactive constituents on psoriasis may be mediated through the suppression of epidermal hyperproliferation and inflammatory responses via modulation of the β-catenin and COX-2 expression.

Collectively, the therapeutic efficacy of FOE in psoriasis management may be attributed to its multifaceted pharmacological properties, including epidermal barrier restoration, normalization of epidermal architecture, and combined antiproliferative and anti-inflammatory activities. Notably, compared with the complete extract or the combined formulation of its three primary components, the individual bioactive components of FOE exhibited reduced therapeutic potential. This observation implies that FOE may contain additional bioactive molecules beyond linalool, 1-octanol, and α-pinene, which may either directly contribute to psoriatic lesion resolution or potentiate the biological activities of the identified compounds. The present study represents an exploratory preclinical investigation solely focusing on the potential mechanism of frankincense in the treatment of moderate-to-severe acute psoriasis. Limitations of this study include the lack of investigation into the effect of the vehicle on the therapeutic outcome against psoriasis, as well as the relatively small sample size and notable intergroup variability, which should be addressed in future research endeavors.

## 4. Materials and Methods

### 4.1. Materials

Frankincense (gum resin from *Boswellia carterii* Birdw., Ethiopia), identified by Prof. Xiaochuan Ye (Hubei University of Chinese Medicine, Wuhan, China), was purchased from Yinpian Factory, Guangzhou Medicine Company (Guangzhou, China). Frankincense oil extract (FOE) used in this study was obtained from previously collected samples (Batch No. 20150313), which are maintained in the specimen repository of the Hepatopharmacology and Ethnopharmacology Department at the School of Pharmaceutical Sciences, South-Central Minzu University. Chemical characterization of the extract was performed via gas chromatography–mass spectrometry, revealing the presence of three principal constituents: linalool (0.0593 mg/mL), α-pinene (0.072 mg/mL) and 1-octanol (0.359 mg/mL) [8].

The chemical reagents employed in this investigation included α-pinene (obtained from Tokyo Chemical Industry, Shanghai Branch, Shanghai, China), linalool (procured from Aladdin Biochemical Technology, Shanghai, China), 1-octanol (supplied by Sinopharm Chemical Reagent Company, Shanghai, China), 5% IMQ cream (Mingxin Pharmaceuticals, Chengdu, China), 0.005% calcipotriol ointment (Leo Pharma, Ballerup, Denmark), 1% tapinarof (Zhonghao Pharmaceutical, Zhongshan, China), 0.1% dithranol ointment (Fangming Pharmaceutical, Heze, China), 0.075% compound dexamethasone acetate ointment (CR Sanjiu; Beijing, China), XAV-939 (Glpbio, Montclair, CA, USA), and dyclonine hydrochloride (Aladdin; Shanghai, China). Immunological reagents consisted of specific primary antibodies targeting COX-2 (manufactured by Cell Signaling Technology, Danvers, MA, USA), β-catenin (produced by ABclonal Biotechnology, Wuhan, China), TRPV3 (sourced from Invitrogen Corporation, Carlsbad, CA, USA), and keratin 10 (provided by Abmart Pharmaceutical Company, Wuhan, China). Histological analysis was performed using H&E staining kits acquired from Nanjing Jiancheng Bioengineering Institute, Nanjing, China.

### 4.2. Animal Models

Male Kunming strain mice, aged six weeks with body weights in the range of 18–22 g, were housed at the Hubei Laboratory Animal Research Center. A psoriasis-like dermatitis model was established in Kunming mice through daily topical application of 62.5 mg IMQ per 3 cm^2^ of dorsal skin surface area over an eight-day period. The mice were euthanized on day 8. The experimental cohort was divided into eight distinct treatment arms, each consisting of five animals: control, model (5% IMQ cream), calcipotriol (vitamin D analog; 20 mg/cm^2^), tapinarof (aryl hydrocarbon receptor agonist; 20 mg/cm^2^), dithranol (antikeratin; 20 mg/cm^2^), dexamethasone (corticosteroid; 20 mg/cm^2^), dyclonine (TRPV3 inhibitor; 0.5 mg/mL, 5 mg/kg/d), and XAV-939 (Wnt/β-catenin signaling pathway inhibitor; 1 mg/mL, 10 mg/kg/d).

The experimental cohort was divided into seven distinct treatment arms, each consisting of five animals: control, model (IMQ control), frankincense oil extract (FOE), linalool (0.0593 mg/mL), α-pinene (0.072 mg/mL), 1-octanol (0.359 mg/mL), or a combined formulation containing all three active ingredients. The dosage concentrations of the individual components were standardized according to their quantitative presence in the FOE preparation. At six-hour intervals post-IMQ administration, each therapeutic compound was uniformly distributed onto the dorsal skin of the assigned mice.

Psoriatic lesion severity was then quantified using the Psoriasis Area and Severity Index (PASI) scoring method, which evaluates three key features: erythema, scaling, and thickening. Each parameter was independently rated on a 4-point ordinal scale: 0, indicating the absence of symptoms; 1, representing mild presentation; 2, corresponding to moderate manifestation; 3, denoting pronounced severity; and 4, reflecting the extreme intensity of features. Overall skin changes were assessed on days 1, 3, 6, and 8.

### 4.3. Spleen-to-Body Weight Index

Throughout the experimental period, body mass measurements were systematically performed for all the groups. Following euthanasia, the splenic tissues were carefully excised from each murine sample and subjected to photographic documentation prior to gravimetric analysis. To evaluate relative organ size, the splenic mass was standardized against total body weight (spleen weight/body weight), yielding an organ index expressed as milligrams of splenic tissue per gram of body mass (mg/g).

### 4.4. Histopathological Analysis of Skin Tissue

The tissue samples from all the groups were harvested and then immediately fixed in a 10% neutral buffered formalin solution. Following standard histological processing, 3 μm sections were prepared and stained with H&E following the protocol provided by the reagent supplier. Microscopic analysis was performed using a Nikon 50i light microscope imaging system (Nikon Corporation, Tokyo, Japan). The histopathology of the skin was evaluated using the Baker scoring method.

### 4.5. Immunohistochemistry

After paraffin removal and rehydration, antigen retrieval was performed on the embedded cutaneous tissue samples using 0.5% pepsin solution for enzymatic digestion. Tissue peroxidase activity was subsequently inhibited through treatment with 3% hydrogen peroxide. The slides were subjected to blocking with 5% BSA at 37 °C for one hour, followed by rinsing with phosphate-buffered saline. Primary antibody incubation was carried out at 4 °C for approximately 16 h using specific antibodies against TRPV3 (1:1000), β-catenin (1:200), COX-2 (1:1000), and keratin 10 (1:50). Chromogenic visualization was achieved through DAB substrate reaction, with nuclear counterstaining performed using hematoxylin. Quantitative image analysis was conducted using the CRi Nuance Multispectral Imaging System (Cambridge Research & Instrumentation, Woburn, MA, USA), which captures spectral data across the 420–720 nm range at 10 nm increments.

### 4.6. Statistical Analysis

The data were analyzed by two-way analysis of variance (ANOVA) supplemented with Bonferroni’s multiple comparison test for post hoc evaluation, establishing a threshold of *p* < 0.05 for statistical relevance. The quantitative results were graphically represented and statistically analyzed via GraphPad Prism version 8.0.1, with the experimental outcomes expressed as arithmetic means accompanied by their standard errors of the mean (SEMs).

## 5. Conclusions

In conclusion, this study highlights FOE and its bioactive constituents as potential therapeutic agents for psoriasis. Our findings suggest that the therapeutic effects may be mediated in part by attenuated TRPV3 expression, elevated K10 expression, and reduced expression of β-catenin and COX-2. These observed changes are associated with anti-inflammatory and immunomodulatory actions, as well as enhanced skin barrier repair. These findings broaden the pharmacological research scope of FOE, establishing it as a promising therapeutic candidate for psoriasis and providing valuable insights into the clinical management of this condition.

## Data Availability

The original contributions presented in this study are included in the article/Supplementary Materials. Further inquiries can be directed to the corresponding authors.

## Supplementary Materials

The supporting information can be downloaded at: https://www.mdpi.com/article/10.3390/ijms27062629/s1.

## Abbreviations

The following abbreviations are used in this manuscript:

FOE	Frankincense oil extract
IMQ	Imiquimod
TRPV3	Transient receptor potential vanilloid 3
K10	Keratin-10
COX-2	Cyclooxygenase-2
