# Supplementary material for: Topical Application of Frankincense Oil Extract Potently Ameliorates Psoriasis-like Dermatitis in Mice via Anti-Inflammatory and Skin Barrier-Protective Effects
Source: Int J Mol Sci. 2026 Mar 13;27(6):2629. doi: 10.3390/ijms27062629 (PMC13026472; doi:10.3390/ijms27062629)
Supplement: Supplementary file 1 [file ijms-27-02629-s001.zip › ijms-4117723-supplementary.pdf]

1. Methods

Quantifying the contributions of therapeutics

Radar charts were created based on seven indicators such as PASI score, pathology score, TRPV3 expression, K10 expression, β-catenin expression and COX-2 expression. The percentage of each coordinate of the radar chart was calculated as: (model group score - treatment group score) / (model group score - blank group score), and the result was expressed as a percentage. The percentage on the coordinate axes indicates the contribution of the therapeutic agent under that metric. The ratio of the polygon connected by the coordinate axes to the area of this radar plot is expressed as the contribution of the therapeutic agent to the mouse model of psoriasis.

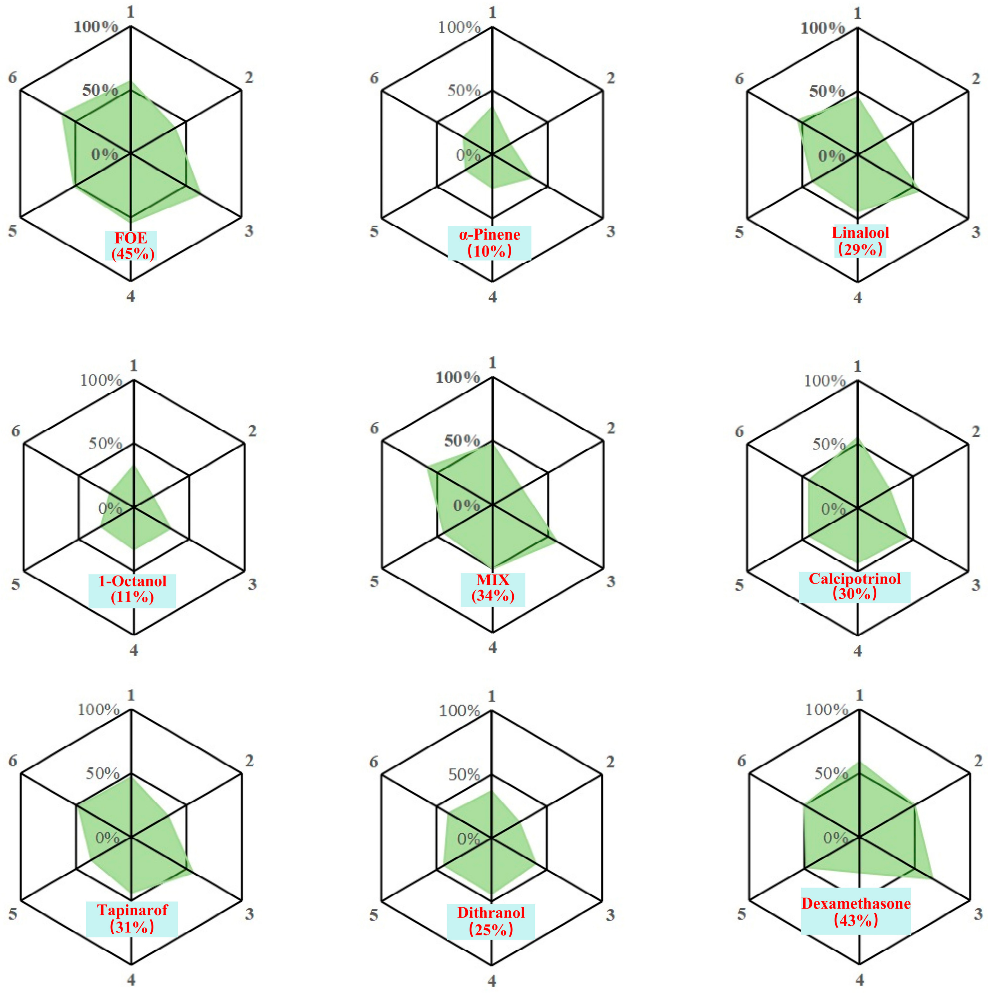

Supplementary Figure S1. Quantitative plot of the improvement of each

therapeutic agent in the mouse model of psoriasis. The meanings of the axes in the graphs are: 1 for PASI score, 2 for pathology score, 3 for TRPV3 expression level, 4 for K10 expression level, 5 for β-catenin expression level, and 6 for COX-2 expression level. The axes are labeled as 100%.
